# Prevention of chemotherapy-induced premature ovarian insufficiency in mice by scaffold-based local delivery of human embryonic stem cell-derived mesenchymal progenitor cells

**DOI:** 10.1186/s13287-021-02479-3

**Published:** 2021-07-31

**Authors:** Eun-Young Shin, Da-Seul Kim, Min Ji Lee, Ah Reum Lee, Sung Han Shim, Seung Woon Baek, Dong Keun Han, Dong Ryul Lee

**Affiliations:** 1 Department of Biomedical Science, CHA University, 335 Pangyo-ro, Bundang-gu, Seongnam-si, Gyeonggi 13488 Republic of Korea; 2 School of Integrative Engineering, Chung-Ang University, 84 Heukseok-ro, Dongjak-gu, Seoul, 06974 Republic of Korea; 3 CHA Advanced Research Institute, CHA Medical Center, 335 Pangyo-ro, Bundang-gu, Seongnam-si, Gyeonggi 13488 Republic of Korea

**Keywords:** Chemotherapy-induced premature ovarian insufficiency, Embryonic stem cell-derived mesenchymal progenitor cells, Bioinspired scaffold, Cell therapy, Oncofertility, Local delivery, Hyaluronic acid

## Abstract

**Background:**

Premature ovarian insufficiency (POI) is one of the most serious side effects of chemotherapy in young cancer survivors. It may not only reduce fecundity but also affect lifelong health. There is no standard therapy for preserving ovarian health after chemotherapy. Recently, administration of embryonic stem cell-derived mesenchymal progenitor cells (ESC-MPCs) has been considered a new therapeutic option for preventing POI. However, the previous method of directly injecting cells into the veins of patients exhibits low efficacy and safety. This study aimed to develop safe and effective local delivery methods for the prevention of POI using two types of bioinspired scaffolds.

**Methods:**

Female mice received intraperitoneal cisplatin for 10 days. On day 11, human ESC-MPCs were delivered through systemic administration using intravenous injection or local administration using intradermal injection and intradermal transplantation with a PLGA/MH sponge or hyaluronic acid (HA) gel (GEL) type of scaffold. PBS was injected intravenously as a negative control. Ovarian function and fertility were evaluated 4 weeks after transplantation. Follicle development was observed using hematoxylin and eosin staining. The plasma levels of sex hormones were measured using ELISA. Expression levels of anti-Müllerian hormone (AMH) and ki-67 were detected using immunostaining, and the quality of oocytes and embryos was evaluated after in vitro fertilization. The estrous cycles were observed at 2 months after transplantation.

**Results:**

The local administration of human ESC-MPCs using the bioinspired scaffold to the backs of mice effectively prolonged the cell survival rate in vivo. The HA GEL group exhibited the best recovered ovarian functions, including a significantly increased number of ovarian reserves, estrogen levels, and AMH levels and decreased apoptotic levels. Furthermore, the HA GEL group showed improved quality of oocytes and embryos and estrous cycle regularity.

**Conclusions:**

HA GEL scaffolds can be used as new delivery platforms for ESC-MPC therapy, and this method may provide a novel option for the clinical treatment of chemotherapy-induced POI.

**Supplementary Information:**

The online version contains supplementary material available at 10.1186/s13287-021-02479-3.

## Background

Advances in anticancer treatment and early detection have led to an increase in the number of young cancer survivors [1]. Chemotherapy, in particular, prolongs survival in patients with cancer, but it may affect the quality of life by inducing long-term adverse effects, such as premature ovarian insufficiency (POI) [2, 3] and permanent infertility. POI is defined as the loss of ovarian function before the age of 40 years [4]. POI is characterized by amenorrhea or oligomenorrhea for at least 3 months, significantly decreased ovarian reserve, hypergonadotropism, abnormally low levels of estradiol, and poor ovarian response to circulating follicular stimulation hormone. Chemotherapy-induced amenorrhea (CIA) depends on the type and dose of the agent, patient’s age, and disease, and the onset rate of amenorrhea ranges from 40 to 68% [5–8]. In addition, CIA may be reversible; in a study by Sukumvanich et al. [9], only 25% females resumed menstruation. Furthermore, 10% patients, especially those who experienced CIA episodes for 2 years, resumed menses, but none had regular menstrual cycles within 3 years after diagnosis. Meirow [8] reported that 34% of female patients experienced POI after chemotherapy, and the type of cancer affected the ovarian insufficiency rate (15% for acute myelocytic leukemia, 44% for non-Hodgkin lymphoma, 32% for Hodgkin’s disease, and 50% for breast cancer). These side effects may not only impair fertility but also lead to bone loss, forgetfulness, mood changes, increased risks of cardiovascular and neurologic diseases, and reduced life expectancy [10–15]. Thus, it is important to consider the prevention of ovarian insufficiency in premenopausal patients with cancer.

Mesenchymal progenitor cells (MPCs) from fetal or adult tissues are being clinically considered a new therapeutic cell source for a variety of diseases. MPCs can secrete multiple cytokines, growth factors, and exosomes containing microRNAs and other molecules, which affect immune modulation, angiogenesis, apoptosis, cell survival, and proliferation [16, 17]. Several studies on MPCs from various tissues have recently been conducted to improve clinical outcomes and overcome POI [18–23]. However, harvesting adult MPCs sometimes requires an invasive procedure that may cause severe side effects, and the culture protocol for MPCs is difficult to standardize. Embryonic stem cell-derived MPCs (ESC-MPCs) are an alternative to adult tissue-derived MPCs because of their high proliferative capacity and easy standardization [24]. Our previous study showed that intravenous (I.V.) injection of ESC-MPCs following chemotherapy can restore ovarian function in mice with cisplatin-injured ovaries [25]. However, this method has some drawbacks, such as the risk of pulmonary embolism and the low rate of injected cells accumulating at the injured site, with many cells sequestered in the spleen, liver, kidney, or lung [26–28]. The poor survival rate after implantation is the most important limitation to MPC therapy because only 1–20% of cells survive after transplantation, which limits their functions [29]. Therefore, we aimed to develop an alternative delivery method for MPC therapy that is minimally invasive, avoids nontarget tissue integration, offers a homogenous cell distribution, and increases the residual cell proportion in vivo.

Numerous types of polymeric scaffolds have been studied as stem cell delivery systems because of their biocompatibility [30–35]. Here, we used two different types of scaffolds as MPC cargo systems: porous poly(D, L-lactide-co-glycolide) (PLGA) sponges containing magnesium hydroxide (Mg(OH)_2_, MH; PLGA/MH sponge) and injectable crosslinked hyaluronic acid (HA) hydrogel (GEL). PLGA has been clinically used as a biomaterial for decades and is approved by the American Food and Drug Administration (FDA) [35]; however, the degradation byproducts lactic acid and glycolic acid can cause an inflammatory response at the implantation site [36–38]. To address this issue, we previously evaluated whether the presence of MH can neutralize the acidic microenvironment formed by the degradation process [39–43]. MH dissolves slightly and sustainably to produce magnesium and hydroxide ions in water, which combine with acidic H^+^ ions generated from the PLGA decomposition byproducts to exert a neutralizing effect in the microenvironment that can reduce inflammation at the implantation site [40]. The main cause of the loss of MPC is cell death, caused by a harsh microenvironment characterized by hypoxia, oxidative stress, and inflammation [44]. Thus, we hypothesized that PLGA/MH sponges, which exert anti-inflammatory effects, are good candidates for improving MPC survival after transplantation.

HA is a major component of the natural extracellular matrix (ECM) in mammalian tissues and has also been approved by the FDA for clinical applications. HA has excellent biological properties, such as biodegradability, biocompatibility, and non-immunogenicity, and provides a three-dimensional microenvironment [41, 42]. Owing to its favorable properties, HA has been researched as a biomaterial scaffold for tissue engineering and regenerative medicine [45–53]. Gel-type HA can be directly injected into specific sites, making it suitable for delivering cells using minimally invasive methods. Moreover, HA gels are suitable for rapid nutrient diffusion and homogenous cell distribution, which might be favorable properties for the therapeutic functions of MPCs against diseases [54].

Several laboratories have used biomaterial scaffolds for in vitro cultivation to grow follicles outside the ovary or to create an artificial ovary [55–57]. To date, follicle-seeded scaffolds have been implanted by invasive methods such as intraovarian microinjection or kidney capsule transplantation [58–60]. In the present study, we evaluated for the first time whether ESC-MPCs can be maintained after subcutaneous delivery with scaffolds and analyzed their effects on the prevention of ovarian insufficiency after transplantation in a cisplatin-induced POI mouse model. We found that locally delivered ESC-MPCs using HA gel and a PLGA/MH sponge could be maintained for more than 4 weeks, and their secretome may help prevent ovarian degeneration and retain female fecundity in the face of chemotherapy-induced apoptotic processes. Our approach may provide a new, simple, and effective method to preserve ovarian function and lifelong health in cancer survivors.

## Methods

### Materials

Injectable crosslinked hyaluronic acid-based hydrogel (HA gel) was obtained from CHA Meditech Co., Ltd. (Gyeonggi-do, Korea). Poly(D, L-lactide-co-glycolide) (PLGA, LA:GA = 50:50, MW 40,000 Da) was obtained from Evonik Ind. (Essen, Germany). Magnesium hydroxide (Mg(OH)_2_, MH), hyaluronidase, and phosphate-buffered saline (PBS, Gibco, Franklin Lakes, NJ) were purchased from Sigma-Aldrich (St. Louis, MO). Dichloromethane (DCM) was purchased from Daejung Chemical (Gyeonggi-do, Korea).

### Scaffold fabrication

Based on our previous publications, PLGA sponge-type scaffolds with 15% MH were fabricated using micro-sized ice particles (100–200 μm) as a porogen. Ice particles were fabricated by spraying deionized water into liquid nitrogen. PLGA was dissolved in dichloromethane, and subsequently, the solution was mixed with 15% MH in a −20 °C cold room. The mixture was fully filled in a Teflon mold (8 mm diameter and 2 mm thickness). The filled mixture was completely lyophilized with a freeze dryer for 2 days. The PLGA sponge-type scaffold was also fabricated with the same procedure without MH.

### Scaffold characterization

The mass loss of HA gel was weighed using cell culture inserts for 24 wells (8 μm pore size) in 1 mL PBS. The sponge was also immersed in 1 mL PBS for 28 days. The percentage mass loss was calculated with the following equation (mass_i_ is the initial mass of the sample, and mass_d_ is the mass of the dried sample):

$$ \mathrm{mass}\ \mathrm{loss}\ \left(\%\right)=\left({\mathrm{mass}}_i-{\mathrm{mass}}_d\right)/{\mathrm{mass}}_i\times 100 $$

The pH changes in 1 mL PBS were measured by a pH meter (Mettler Toledo, OH). The morphology of each scaffold was analyzed by scanning electron microscopy (FE-SEM; Hitachi S-4800) after complete drying with a freeze dryer. The thermal properties of each scaffold were measured by a thermogravimetric analyzer (TGA) (PerkinElmer, MA). The temperature went from room temperature to 800 °C at a heating rate of 10 °C/min under a nitrogen atmosphere. The mass-change-versus-temperature curves were recorded. A derivative form of TGA (DTG) was obtained using the differential of TGA values. The chemical bonding of each scaffold was characterized by attenuated total reflection-Fourier transform infrared (ATR-FTIR) spectra (PerkinElmer) in the range of 400–4000 cm^−1^, with a spectral resolution of 4 cm^−1^. The rheological properties of HA hydrogels (50% and 100%) were analyzed using a stress-controlled rheometer from Anton-Paar (Graz, Austria), which was equipped with parallel-plate geometry at a diameter of 25 mm. The gap was fixed at 1000 μm. The frequency sweep was carried out with a frequency range of 0.1–10 Hz to determine the linear viscoelastic (LVE) zone for the hydrogel.

### Differentiation and cultivation of human ESC-MPCs

Differentiation and characterization of human ESC-MPCs were performed as described in our previous reports [24, 25, 61]. Briefly, the human ESC line CHA-hES15 (Korea Stem Cell Registry No. hES12010028) was detached by a mechanical method using a glass pipette (Corning, Corning, NY) and cultured in a Petri dish (Corning) for embryoid body (EB) formation. Fourteen days after EB formation, the cells were attached to culture dishes and outgrowth cells were maintained in Dulbecco’s modified Eagle’s medium (DMEM)/low glucose (Hyclone, Logan, UT) supplemented with 10% fetal bovine serum (FBS, Gibco), 0.1 mM beta-mercaptoethanol (Invitrogen, CA), 1% nonessential amino acids (NEAAs, Gibco), and 1% penicillin–streptomycin (P/S, Gibco). Sixteen days after EB attachment, the outgrowth cells were subcultured and further maintained in DMEM/F12 medium (Gibco) supplemented with 10% FBS (Gibco), 1% NEAA (Gibco), 1% P/S (Gibco), and 0.1 mM mercaptoethanol (Invitrogen). These cells were defined as human ESC-MPCs and were used in this study. The characteristics of human ESC-MPCs were described in our previous reports [24, 25, 61].

### In vitro cell biocompatibility test

Fabricated PLGA and PLGA/MH sponge-type scaffolds were serially rinsed in 70% ethanol, DW, and phosphate-buffered saline (PBS) for hydration. After hydration, 5 × 10^6^ human ESC-MPCs were seeded onto the scaffolds. After 1 h, the medium was added and incubated to adhere to the scaffold overnight and then used for testing the biocompatibility.

An injectable crosslinked HA-based hydrogel (HA gel) was used without dilution for obtaining 100% HA gel but was diluted with PBS for obtaining 50% HA gel. Then, each gel was mixed with 5 × 10^6^ human ESC-MPCs using a 21 G syringe (Kovax-1 mL syringe; Korea Vaccine Co., Ltd., Gyeonggi-do, Korea).

For live/dead staining, cells were incubated with 2 μM calcein AM and 4 μM EthD-1 in PBS for 20 min and subsequently imaged using a confocal microscope (Zeiss LSM 880, Carl Zeiss AG, Oberkochen, Germany).

### Animal experiments

Six-week-old ICR female mice were purchased from KOATECH (Gyeonggi-do, Korea) and used to induce a cisplatin-induced premature ovarian insufficiency (CIP) mouse model. Mice were intraperitoneally injected with cisplatin (2.0 mg/kg) or saline for 10 days. Saline-injected mice were used as WT control (Normal group) and cisplatin-injected mice were used as the CIP model.

Mice were randomly assigned to six groups: POI, intravenous injection (I.V.), intradermal injection (I.D.), HA gel type of scaffold (GEL), PLGA/MH sponge type of scaffold (Sponge), and WT. The experimental settings and groups are shown in detail in Fig. 2A. The body weight of each animal was checked every other day after transplantation. Scaffold samples were collected at days 0, 7, and 28, and ovaries and plasma were collected at day 28 after transplantation.

### Cell transplantation

On day 11 of cisplatin administration, cisplatin-induced POI (CIP) mice were transplanted with human ESC-MPCs (passages 8–10; CHA Stem Cell Institute, Korea) using four different methods. The cells were detached from the dish with 0.125% trypsin/EDTA (Gibco) and centrifuged with PBS three times to remove the medium. Then, they were diluted to 5 × 10^6^/220 μL per mouse with PBS. The mice were anesthetized using 2,2,2-tribromoethanol (Avertin, Sigma-Aldrich), and human ESC-MPCs were delivered through systemic administration using I.V. or local administration using intradermal injection (I.D.) on the dorsal part of the nearby ovaries. PBS was injected intravenously as a negative control.

For transplantation of human ESC-MPCs with PLGA/MH sponge, the CIP mice were anesthetized with avertin, and a 1-cm longitudinal incision was made on the dorsal part on the integument such that the nearby ovaries of each mouse were exposed to the endodermis. The scaffold with cells was then transplanted intradermally using forceps, and the incision was closed by suturing.

For transplantation of human ESC-MPCs with HA gel, the CIP mice were anesthetized with avertin. Then, 100 μL of cells and HA gel mixture (5 × 10^6^ cells/50 μL PBS and 50 μL HA gel) were injected into the dorsal part of the ovaries of mice using a 21 G syringe.

The experiments were performed in quadruplicate (total *n* = 14 in the normal control; total *n* = 24 in the POI group; total *n* = 25 in the I.V. group; total *n* = 22 in the I.D. group; total *n* = 24 in the Sponge group; and total *n* = 22 in the GEL group).

### Ovarian follicle counting

The mouse ovaries were harvested at 4 weeks after cell transplantation. The left ovary was fixed in 4% formaldehyde for 3 days and embedded in paraffin. Each ovary was serially sectioned to 5 μm thickness and stained with hematoxylin and eosin to evaluate follicle growth. Follicles (primordial, primary, secondary, antral follicles and zona pellucida remnants) were classified and counted. The percentage of follicles at each stage was calculated and compared between groups. The follicles were classified as previously described [62].

### Detection of AMH and proliferation in ovarian tissue

To detect the AMH and proliferation levels of ovarian tissue, tissue sections were deparaffinized and blocked with a DAKO blocking solution (Dako North America, Carpinteria, CA) for 2 h at room temperature. Sections were incubated overnight with AMH and Ki-67 (Abcam, Cambridge, MA) antibodies at 4 °C. The secondary antibodies were Alexa 555-conjugated anti-mouse for AMH, Alexa 488-conjugated anti-rabbit for Ki-67, and DAPI staining for the detection of nuclei.

### Enzyme-linked immunosorbent assay (ELISA)

Plasma was harvested to evaluate the levels of E2 and FSH by using ELISA kits (MyBioSource) according to the manufacturer’s instructions. Briefly, a 50-μL plasma sample was diluted with sample buffer and added to each well. Then, a 50-μL blank, standard, or plasma sample was added to each well. Fifty microliters of biotin-labeled antibody working solution was added to each well. The microplate was covered with a plate sealer and incubated for 45 min at 37 °C. The cover was removed and washed with wash buffer 3 times. Thereafter, 50 μL of HRP-streptavidin conjugate reagent was added to each well and incubated for 30 min at 37 °C. The plate was washed with wash buffer 5 times. Then, 90 μL of TMB substrate was added to each well and incubated for 15 min at 37 °C in the dark. Fifty microliters of stop solution was added to each well. Finally, the light absorbance (O.D. 450) was measured and recorded by a microplate reader (Varian Company, Australia).

### Western blot analysis

Harvested ovaries were homogenized in PRO-PREP^TM^ Protein Extraction Solution (17081, Intron Biotech, Gyeonggi-do, Korea). After centrifugation at 13000 rpm for 5 min, the supernatant was transferred to a fresh tube. The protein was diluted with 2× sample buffer (4% sodium dodecyl sulfate (SDS), 20% glycerol, 130 mM Tris-Cl, and 0.02% bromophenol blue). The diluted protein was loaded in a 10% gel and separated by electrophoresis. Separated proteins were transferred to a polyvinylidene difluoride membrane (Bio-Rad, Hercules, CA). The membrane was blocked with 5% BSA (Bio-Rad) and incubated with primary antibody against PARP (Cell Signaling, Danvers, MA). The membrane was then treated with HRP-conjugated anti-rabbit secondary antibody (Bio-Rad). The specific signal was visualized by enhanced chemiluminescence (Amersham Hyperfilm ECL, GE Healthcare, Pittsburgh, PA). Stripped membranes were reprobed with anti-α-Tubulin (Cell Signaling). Visualized bands were analyzed by densitometry using NIH Imaging J software. The experiments were repeated more than three times, and the results are presented as the fold change ± SE.

### Oocyte collection and fixation

Female mice were superovulated by intraperitoneal injection of 5 IU of pregnant mare serum gonadotropin (Sigma-Aldrich), followed 48 h by 5 IU of human chorionic gonadotropin (hCG; Sigma-Aldrich). Cumulus oocyte complexes (COCs) were collected in M2 (Sigma-Aldrich) medium at 14 h after hCG injection. For immunofluorescence staining, cumulus cells were removed with 0.1% hyaluronidase (Sigma-Aldrich) and then washed twice in the M2 medium. The zona pellucida of denuded eggs were removed by brief (approximately 15 s) incubation in acidified Tyrode’s solution (Sigma) and then washed twice in the M2 medium. Zona-free oocytes were washed with PBS containing 0.1% polyvinyl alcohol (Sigma) for 15 min and then fixed in 4% paraformaldehyde for 30 min. Fixed oocytes were washed twice in PBS with 0.1% BSA (Sigma), permeabilized with 0.2% Triton X-100 in PBS overnight, and kept at 4 °C until use.

### In vitro fertilization and embryo development

Epididymal sperm from 6- to 8-week-old male CD-1 mice were collected in 500 μL of HTF medium (Millipore) and allowed to capacitate for 1 h before use. COCs were inseminated for 4–5 h in a 50-μL drop of HTF medium and then the fertilized embryos were quickly washed in drops of KSOM medium (Millipore) by using a pasture pipette to remove unbound sperm and cumulus cells. Embryos were cultured in a KSOM medium in a humidified 5% CO2 atmosphere. The 2-cell rate was recorded on 24 h after IVF, and the number of blastocysts was counted on day 5. Experiments were independently repeated for 4 times with the same condition (total *n* = 17 in the normal control; total *n* = 24 in the POI group; and total *n* = 22 in the GEL group).

### Residual MPC detection

Immunohistochemistry (IHC) was performed with the antibody to stem121 which reacts specifically with a cytoplasmic protein of human cells to detect residual MPCs in the scaffolds. The scaffolds were carefully collected 1 and 4 weeks after implantation, fixed with 4% formaldehyde for 7 days at 4 °C, and serially sectioned using a microtome (5 μm thickness). The collected scaffolds were embedded in paraffin, deparaffinized with xylene, and rehydrated with serially diluted ethanol in the same way as above. The slides of sectioned scaffolds were incubated with a human cytoplasmic stem121 antibody (Takara bio, Japan) at 4 °C overnight. The secondary antibody was GFP-conjugated anti-mouse (Invitrogen) to detect stem121. Nuclei were stained with DAPI.

Human genomic DNA detection on scaffolds taken back from 1 and 4 weeks implanted scaffolds were performed. Human genomic DNA was extracted by the AccuPrep® Genomic DNA Extraction Kit (Bioneer, Daejeon-si, Korea). The implanted HA gel was hydrolyzed with hyaluronidase (50 U/scaffold) for 15 min at 37 °C and the sponge was cut into small pieces following the manufacturer’s protocol with tissue extraction. Fifty nanograms of genomic DNA template was used in PCR, and the primer sequences for human SRY and hALU were used to detect human cells. GAPDH primers that could detect both human and mouse genes were used as the control gene. The primer sequences were hSRY-F (GTAAAGGCAACGTCCAGGATAGAG) and hSRY-R (GCATCTAGGTAGGTCTTTGTAGCC); h,mGAPDH-F (TCAAGAAGGTGGTGAAGCAGG) and h,mGAPDH-R (CACATACCAGGAAATGAGCTT); and hALU-F (GGAGGCTGAGGAGGAGAA) and hALU-R (C-GGAGTCTCGCTCTGTCG CCCA).

### Growth factor array

The growth factor array was performed with a RayBio Human Cytokine Antibody Array (Human Growth Factor Array G1, RayBiotech, Inc., GA). The human ESC-MPCs were cultured with DMEM/F12 containing 0.2% FBS for 6 and 24 h. The conditioned medium was purified with a 0.2-μm syringe filter, and DMEM/F12 containing 0.2% FBS was used as a negative control. The array was performed following the manufacturer’s protocol. Subsequently, the slide was imaged in a GenePix 4000B scanner (Molecular Devices, CA). We sorted significantly overexpressed factors by fold change value (|fold change| > 1.5) and adjusted *p*-value (FDR < 0.01). Gene Ontology and KEGG pathway analyses were performed with EnrichR [63, 64].

### Estrous cycle analysis

Vaginal smears were collected on glass slides in 100 μL of PBS at 9:00–10:00 every morning for 2 weeks. After air drying on a warm plate, the samples were stained with hematoxylin and eosin and then washed and dried. The stages of the estrous cycle were determined by analyzing the proportion of cell types, including epithelial cells, cornified cells, and leukocytes [65]. Consistent cycles of proestrus (Pro), estrus (Est), metestrus (Met), and diestrus (Di) repeated every 4–5 days were termed the “regular estrous cycle” in mice [66]. Irregular estrous cycles were defined as when the mice had at least one prolonged estrous cycle (more than 5 days) until the end of the observation period. The experiments were repeated three times (total *n* = 23 in the normal control; total *n* = 27 in the POI group; and total *n* = 26 in the GEL group), and the results are expressed as the mean ± SE.

### Statistical analysis

All experiments were repeated at least three times. The results are shown as the means ± standard error of the mean (SEM). Statistically significant differences were evaluated by one-way ANOVA with Duncan’s post hoc test using SPSS ver. 18 software (SPSS Inc., Chicago, IL). Embryo developmental rates were analyzed by Student’s *t* test in GraphPad Prism 7.0 software (GraphPad Software, Inc., CA). *p* values < 0.05 were regarded as statistically significant.

## Results

### Design of scaffolds for improving the survival of MPCs after transplantation

The poor survival rate of cells after transplantation and safety issues are major obstacles to MPC therapy. In addition, gradual cell loss after implantation may be caused by a lack of supporting structures; hence, to overcome these limitations, we propose a local MPC delivery system using two different types of scaffolds: PLGA sponge and HA gel [67]. We selected clinically applicable scaffolds that are approved for other tissue engineering applications, such as dermal fillers and drug delivery products [35, 68].

**Fig. 1:**
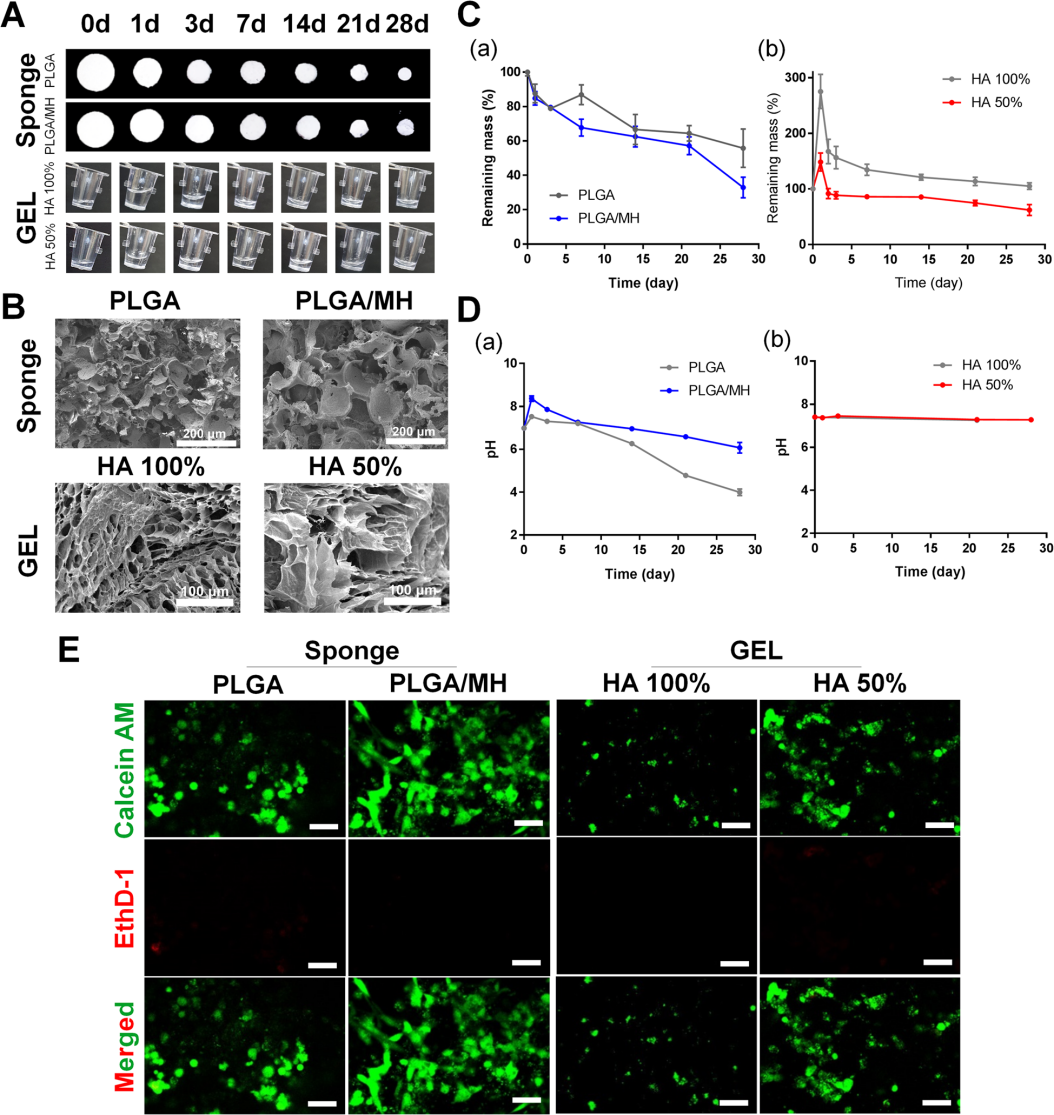
Scaffold characterization; PLGA sponge, and HA gel. **A** Optical images of the two types of scaffold. **B** Scanning electron microscopic images of each scaffold (× 200 magnification, scale bar = 200 μm). **C** Percentage of remaining mass of the scaffolds and **D** pH changes in scaffolds during in vitro degradation at 37 °C for 28 days (n = 3). **E** Live and dead staining images before implantation (scale bar = 100 μm)

Figure 1A shows the optical images of each scaffold during the degradation process under physiological conditions (pH 7.4, 37 °C) for 4 weeks. The initial enlarged morphology of each scaffold was analyzed using scanning electron microscopy (Fig. 1B). The PLGA and PLGA/MH sponges had a porous structure that enhanced the inner capacity for cell encapsulation. Compared with the 100% HA gel, the dehydrated 50% HA gel images seemed to have a loose structure because of the reduced concentration of HA (diluted to 50% with PBS).

Because MH can dissolve in the presence of weak acid although it has low water solubility (K_sp_ = 5.61 × 10^12^) [41], the mass loss of the PLGA/MH rapidly decreased from day 7 onward, indicating that MH neutralized the acidic environment (Fig. 1C (a) and D (a)). HA can retain water; therefore, the initial mass of the HA gel was too high [69]. To reduce the initial swelling and give HA the capacity to mix easily with cells, we diluted it with PBS at a ratio of 1:1. The diluted HA gel was reduced to 53.9% (from 275.5 to 148.5%, Fig. 1C (b)) of the initial mass gain in swelling behavior on day 1. Additionally, there was no significant change in the pH value with the HA gel, as expected (Fig. 1D (b)). Next, the presence of MH and its proportion in the PLGA sponge were confirmed using attenuated total reflection-Fourier transform infrared spectroscopy (ATR-FTIR, Figure S1 (a)) and thermogravimetric analysis (Figure S1 (b)). The HA gel was scanned using ATR-FTIR, and infrared peaks were observed at 3420, 2918, 1602, 1413, and 1033 cm^−1^ (the spectrum of standard HA) [70]. The thermogram of the HA gel demonstrated that the decomposition temperature (T_d_) was 72.81 °C and the residual weight was 29.38% at 800 °C. Additionally, the dynamic moduli of the HA gel were analyzed using the rheometric method, which showed that the storage modulus (G‘), loss modulus (G“), and loss tangent (tanδ) values of the 50% HA gel were lower than those of the 100% HA gel (Figure S2).

To confirm the biocompatibility of each scaffold before implantation, we evaluated the viability of MPCs on scaffolds using live/dead staining (calcein AM and ethidium homodimer-1). Calcein AM is converted to green fluorescent calcein in the cytosol of live cells [71]. Based on fluorescence images (Fig. 1E), MPCs were more stretched in the presence of MH in the sponge (PLGA/MH) than in the presence of PLGA alone. Some dead EthD-1-positive cells were observed on the PLGA-only sponge. It has previously been observed that cell morphology can be controlled by the viscosity of substrates and that gel stiffness can affect cell proliferation [72, 73]. The cells in the 50% HA hydrogel showed better spreading and increased cell number because 50% HA had a lower viscosity (Figure S2). In this regard, we speculated that the PLGA/MH sponge (Sponge) and the 50% diluted HA gel (GEL) are good candidates for improving MPC survival after transplantation because they provide a physical structure to support cell adhesion and reduce anoikis.

### Analysis of paracrine factors in conditioned medium (CM) derived from human ESC-MPCs (in vitro study)

Previous studies demonstrated that secreted paracrine factors from transplanted adult MPCs can restore ovarian function and structure [21, 22, 74, 75]. However, the effect of secretomes from transplanted ESC-MPCs on POI remains unknown. To test this possibility, the paracrine factors derived from ESC-MPCs in CM were analyzed using a human growth factor array to examine 41 human growth factors (Figure S4). Next, we screened for ESC-MPC proteins with a fold change greater than two in CM over DMEM/F12, and the results showed that 12 growth factors were significantly higher than those in the basal medium (*p* < 0.05, Figures S4C and D). The selected paracrine factors secreted by ESC-MPCs were CSF2, CSF3, insulin-like growth factor binding protein (IGFBP) 1, IGFBP2, IGFBP3, IGFBP4, IGFBP6, vascular endothelial growth factor (VEGF) A, fibroblast growth factor (FGF) 2, platelet-derived growth factor (PDGFA), and PDGF receptor alpha (PDGFRα). According to Gene Ontology enrichment analysis by EnrichR, the 12 proteins were classified into five groups, which might play important roles in ESC-MPC-mediated repair of ovarian injuries, such as by participating in wound healing, regulation of cell death and apoptosis, DNA replication, cell differentiation, and regulation of cell proliferation (Figure S4E). Furthermore, KEGG pathway analysis of the selected proteins indicated that ESC-MPC paracrine factors were mainly related to the PI3K-AKT signaling pathway, which is involved in cell proliferation and survival (Figure S4F) [76, 77]. Their roles in antiapoptosis, cell cycle progression, angiogenesis, DNA repair, metabolic processes, and protein synthesis may also contribute to the rescue of ovarian functions in POI [76, 77].

### Stability of scaffolds and detection of residual ESC-MPCs after transplantation (in vivo study)

To examine whether scaffolds can improve the survival rate of ESC-MPCs after transplantation, we detected residual cells from implanted scaffolds. In particular, we focused on a simple delivery method that involves implantation into the intradermal site on the back. Because this site is easily accessible, local administration of MPCs to the injured site may lead to cell death and secondary damage caused by high-density cell administration. As a result, both scaffolds remained in the implanted site until 4 weeks after transplantation (Figures S5A, C, and D). Moreover, human SRY gene expression was detected only in the Sponge group until 4 weeks after transplantation, but a human-specific Alu sequence was detected at all tested time points in both groups (Figure S5B). Interestingly, the stem121 antibody, which reacts specifically with a cytoplasmic protein of human cells, was also found in both scaffolds at 1 and 4 weeks after implantation (Figures S5C and D). Our previous study showed that intravenously injected human ESC-MPCs could be detected in the ovarian tissue on day 3 and only in the spleen on day 7, which is consistent with the findings of other studies showing a rapid decrease 1 week after transplantation [25, 78]. Thus, the most interesting aspect of this result is that the scaffolds improved the survival rate of human ESC-MPCs after transplantation, and the cells resided in vivo until 4 weeks after administration. This is likely because locally delivered human ESC-MPCs with scaffolds could survive longer; thus, they might produce secretomes that would be therapeutic for ovarian injuries for a long time.

### Establishment of a cisplatin-induced POI model and transplantation of human ESC-MPCs restore the body weights of CIP mice

**Fig. 2:**
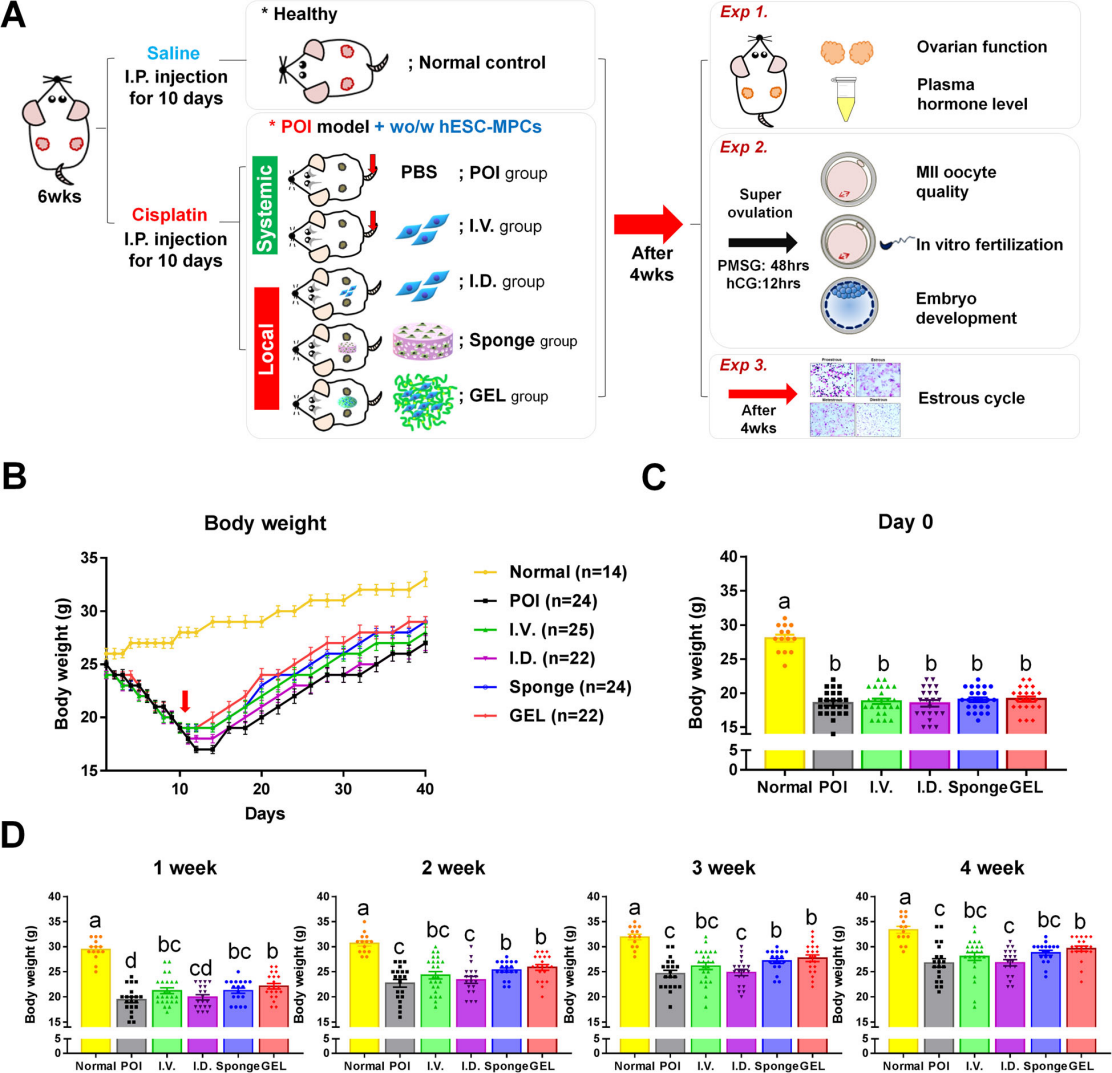
Schematic illustrations of the experimental design and changes in body weight of mice. **A** Experimental scheme for in vivo experiments. **B** Body weights were measured daily during cisplatin administration and then measured every other day after implantation of human MPCs. **C** Body weights of the different groups were the same on the day of transplantation. **D** Body weights at 1, 2, 3, and 4 weeks after cell therapy. Different superscript letters indicate a significant difference (*p* < 0.05); Normal, normal control group; POI, premature ovarian insufficiency; I.V., intravenous; I.D., intradermal; Sponge, PLGA sponge type of scaffold; GEL, HA gel type of scaffold

To evaluate the best administration method for improving POI with transplanted ESCs-MPC, we first attempted to build a CIP model by first administering cisplatin daily for 10 days. On day 11 of cisplatin administration, human ESC-MPCs were delivered through systemic administration using I.V. (I.V. group) or local administration using I.D. (I.D. group) and intradermal transplantation with the PLGA/MH sponge (Sponge group) or HA gel (GEL group)-type scaffolds. PBS was injected intravenously as a mock control treatment (POI group) (Fig. 2A). Although a significant loss of body weight was observed in all CIP mice (*p* < 0.05) compared with normal control mice on the day of transplantation, there was no significant difference between the five groups (POI, I.V., I.D., Sponge, and GEL) (Fig. 2C). At 1 week after transplantation, the I.V., Sponge, and GEL groups had significantly recovered their body weight compared with the POI group, but the I.D. group showed no significant differences in body weight compared with the POI group. At 2 weeks after therapy, the body weight of the I.V. group was similar to that of the POI group. In contrast, compared with the POI group, the Sponge and GEL groups showed significantly increased body weight until 3 weeks after transplantation. Most importantly, the body weight of the GEL group was markedly higher than that of the POI group during the study period (Fig. 2B, D). These results indicate that the local transplantation of human ESC-MPCs with scaffolds can exhibit a higher increase in body weight than that exhibited by the injection of cells without scaffolds in CIP mice.

### ESC-MPC therapy restores ovarian structure and function in CIP mice

**Fig. 3:**
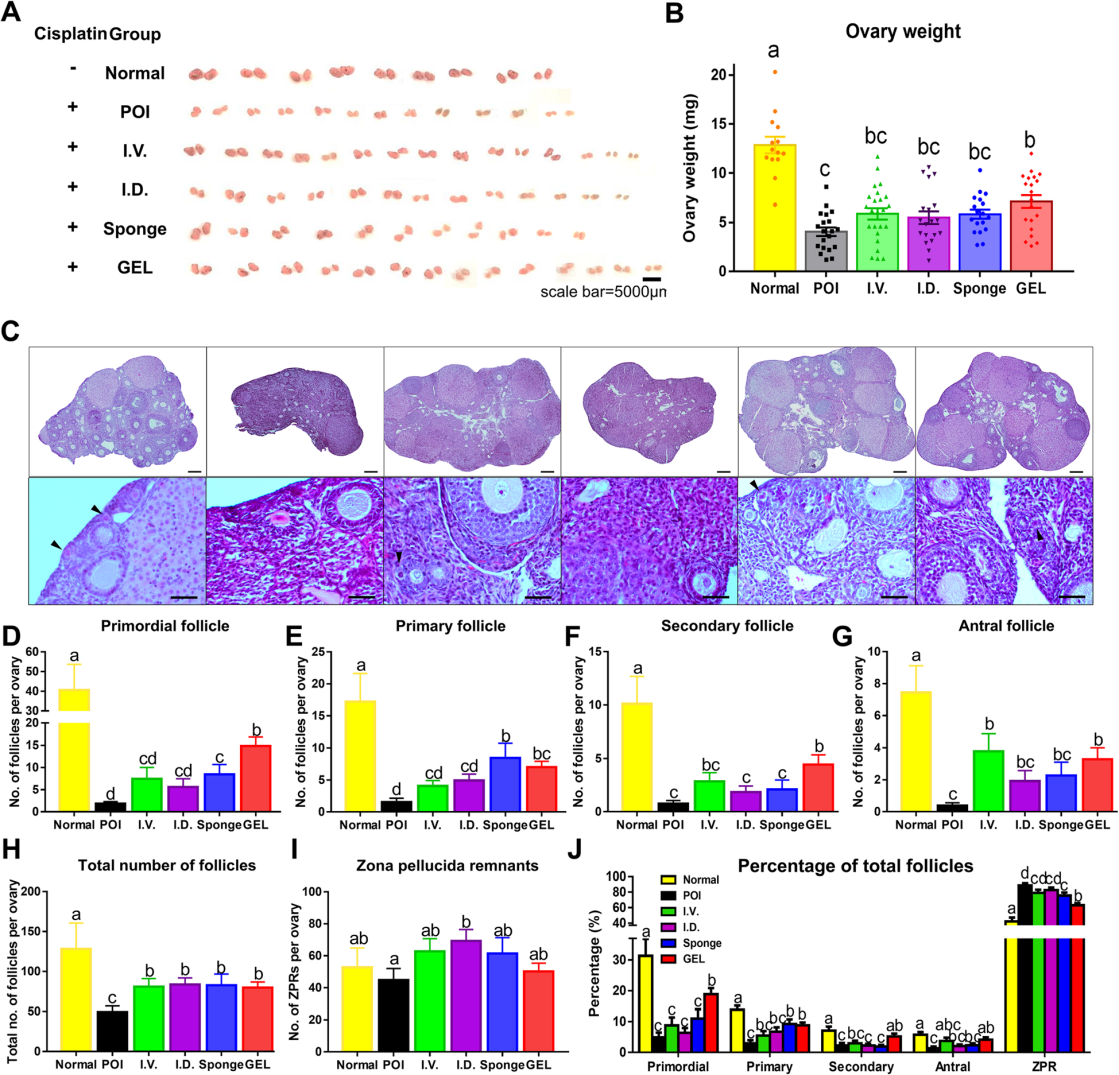
Recovery of the ovarian structure by transplantation of human ESC-MPCs in the CIP model. **A** Gross morphology of ovaries. Scale bar = 5000 μm. **B** Weight of ovaries was measured at 4 weeks after cell therapy. **C** Ovarian histology was analyzed by H&E staining. Scale bar = 200 or 25 μm. The arrow heads indicate primordial follicles. **D**–**G** The number of follicles at different stages (**D**, primordial; **E**, primary; **F**, secondary; **G**, antral) was counted and compared in the six groups at 4 weeks after human ESC-MPC therapy. **H** The number of zona pellucida remnants per ovary. **I** Total number of follicles per ovary. **J** Percentage of follicles at different stages per ovary. The different superscript letters indicate significant differences (*p* < 0.05); Primordial, primordial follicle; Primary, primary follicle; Secondary, Secondary follicle; Antral, antral follicle; ZPR, zona pellucida remnant

To investigate whether the local delivery of human ESC-MPCs can restore ovarian function in POI, we first compared the changes in ovarian size and ovarian weight among the six groups (normal, POI, I.V., I.D., Sponge, and GEL). Examination of the gross morphological appearance revealed that all cell transplantation groups showed improved ovarian size compared with the POI group (Fig. 3A). Although the injection of cisplatin resulted in a significant decrease in ovary weight, the GEL group had a significantly greater ovarian weight than that of the other groups (I.V., I.D., Sponge, and POI) (*p* < 0.05; Fig. 3B). Next, we evaluated the histological changes in the ovaries after the ESC-MPC therapies (Fig. 3C). The numbers of primordial, primary, secondary, antral, and total follicles decreased significantly in all CIP mice compared with those in the normal control group. However, the ESC-MPC group had more total follicles than those in the POI group. Moreover, the numbers of primordial follicles, which represent ovarian reserve, and follicles at various other stages were significantly higher in the GEL group than in the POI groups (*p* < 0.05; Fig. 3D). However, there were no significant differences in the numbers of zona pellucida remnants (ZPRs), which are considered atretic follicles, in most CIP mice compared with the control group (*p* < 0.05; Fig. 3D–I). To exclude the possibility that the difference in contents occurred because of the difference in ovarian volume, the ratio of each follicle number was normalized to the total number of follicles and compared. Only the GEL group had a significantly higher proportion of primordial follicles (18.9 ± 2.0%) than that in the POI group (11.0 ± 3.1%) (*p* < 0.05; Fig. 3J). Interestingly, most follicles in the POI group were ZPRs (88.6 ± 2.9%); in contrast, the GEL group had a significantly lower proportion of ZPRs (*p* < 0.05; 63.0 ± 2.9%) than that in the other groups (I.V., 79.1 ± 3.9%; I.D., 82.7 ± 3.2%; and Sponge, 82.7 ± 3.2%). These results suggest that compared with the other treatments, GEL treatment improved the therapeutic effects of human ESC-MPCs (reducing ovarian injury) in CIP mice.

### Local transplantation of human ESC-MPCs with HA gel reduces ovarian damage and improves ovarian functions in CIP mice

**Fig. 4:**
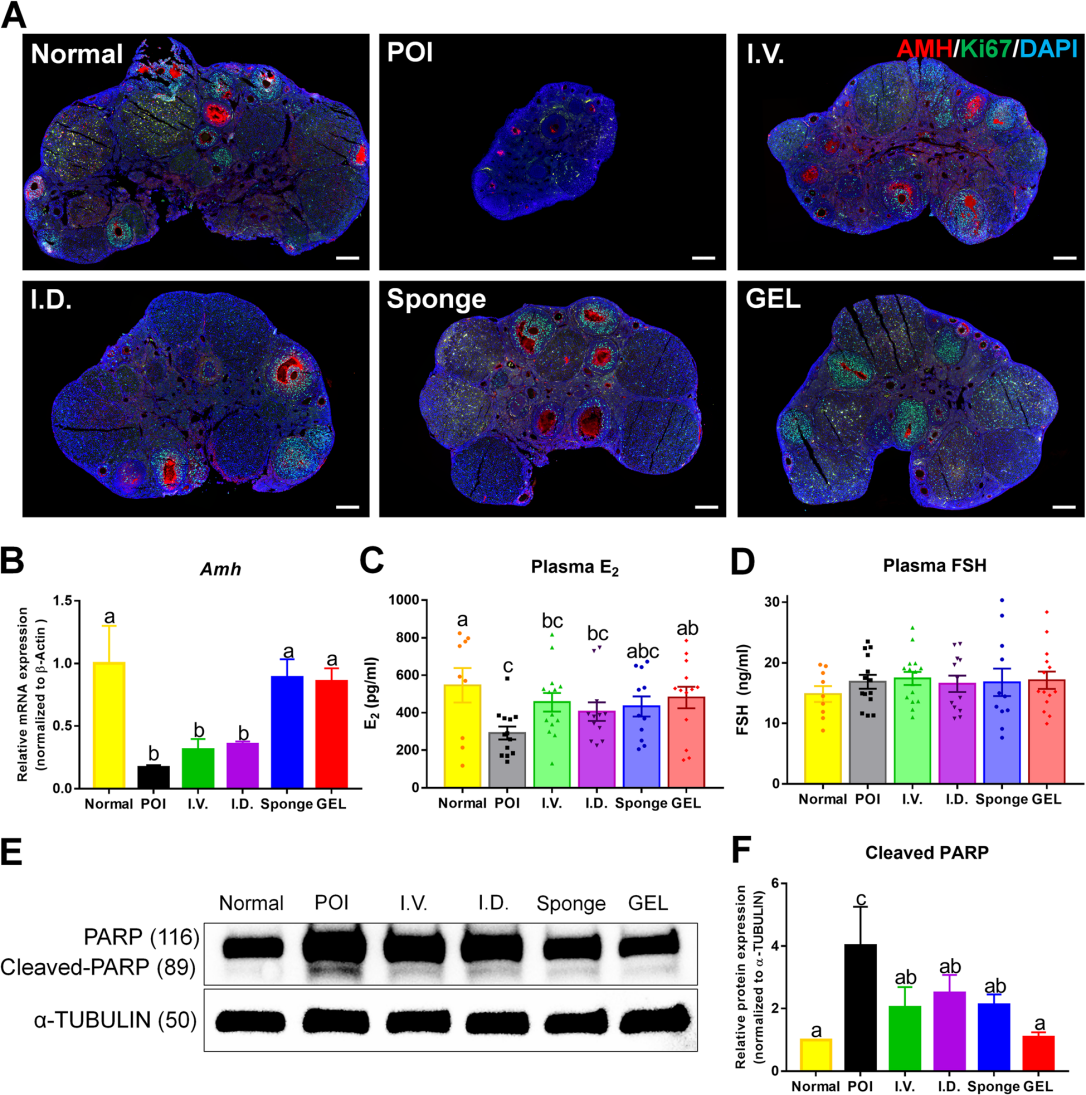
Human ESC-MPC therapy reduces ovarian damage in CIP mice. **A** Immunofluorescence for AMH (red) and Ki-67 (green). DAPI (blue) represents follicle growth and granulosa cell proliferation in the ovary. Scale bar = 200 μm. **B** Relative mRNA expression by qRT-PCR analysis for Amh normalized to β-actin. Fold changes were measured by the 2^−ΔΔCT^ method, n = 3. Serum levels of E2 (**C**) and FSH (**D**). **E** Apoptosis levels in ovaries were evaluated by the expression levels of cleaved PARP using western blot analysis. **F** Western blot results with relative protein levels of cleaved PARP controlled to α-TUBULIN calculated from three different blots. The different superscript letters indicate significant differences (*p* < 0.05); Amh, anti-Müllerian hormone; E2, estrogen; FSH, follicular stimulation hormone

Anti-Müllerian hormone (AMH) is secreted by growing follicles and is a negative regulator of dormant follicle activation [79]. Chemotherapy can trigger dormant follicle activation through low levels of AMH resulting from chemotherapy-induced apoptosis of growing follicles [80–82]. Ovarian expression of AMH was significantly reduced in the POI group but rescued by transplantation of ESC-MPCs. The level of ki67, a marker of proliferating cells, was increased in all cell transplantation groups compared with that in the POI group (Fig. 4A). In addition, the qPCR results demonstrated that the mRNA levels of *Amh* in ovaries were significantly higher after local administration of scaffolds (Sponge and GEL groups) than after other treatments (*p* < 0.05, Fig. 4B). Furthermore, the concentration of estrogen (E2) in the plasma of mice in the GEL group was also significantly higher than that in the POI group (*p* < 0.05, Fig. 4C), whereas the plasma FSH concentration was not significantly different between the groups (*p* < 0.05, Fig. 4D). To further test the apoptotic levels of the ovaries, the expression of cleaved PARP was measured in cisplatin-injured ovaries using western blotting. Consequently, cleaved PARP levels were significantly lower in the GEL group than in the POI group (*p* < 0.05, Fig. 4E, F). These results indicate that the transplantation of human ESC-MPCs with GEL can reduce the apoptotic activity in chemotherapy-damaged ovaries and can retain ovarian functions in CIP mice.

### Local transplantation of human ESC-MPCs using HA gels can retain the quality of oocytes and the developmental potential of IVF embryos from CIP mice

**Fig. 5:**
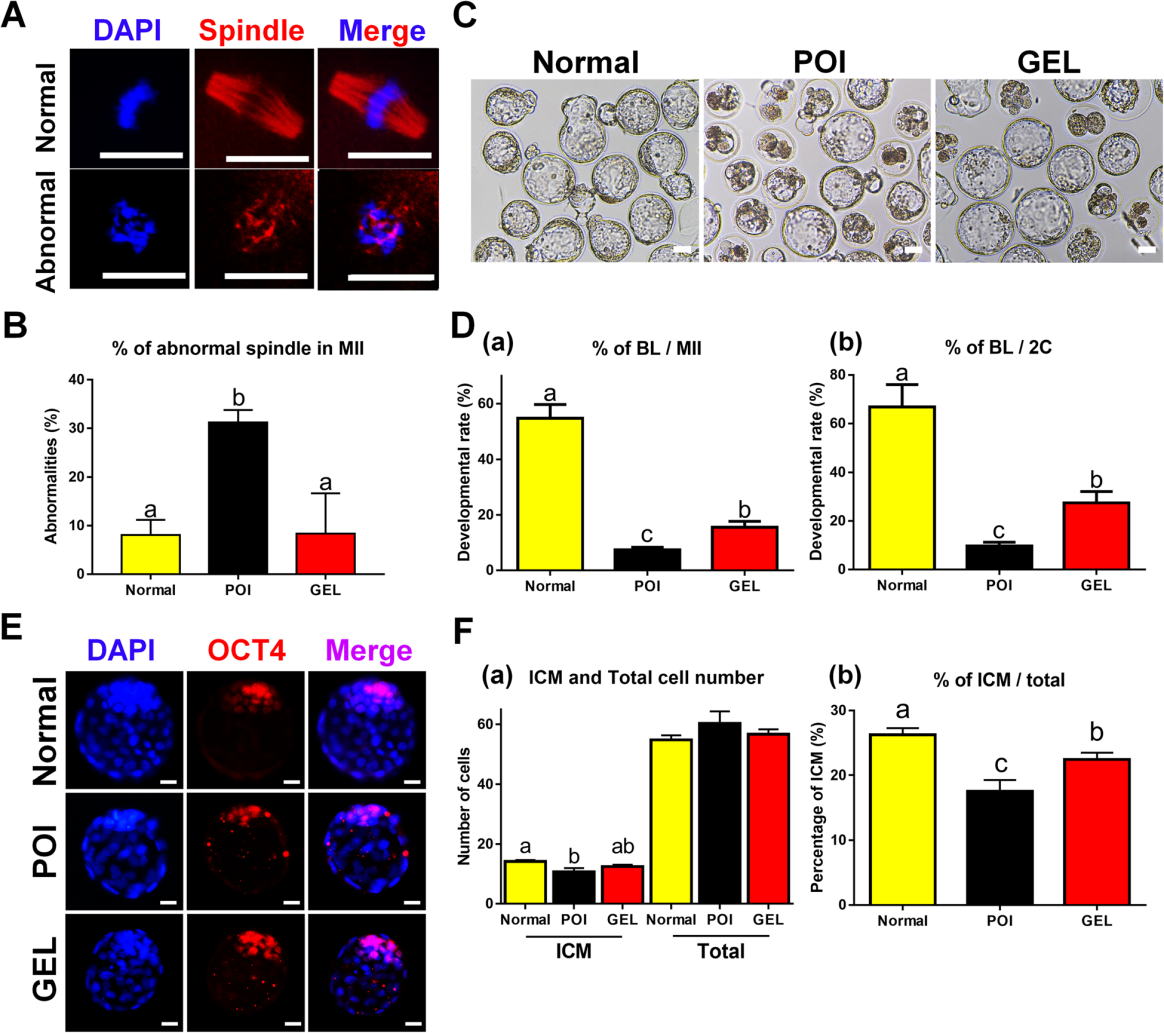
Transplantation of human ESC-MPCs with HA gel improves the quality of oocytes and the development of embryos. **A** Representative images of spindle morphology in normal and abnormal MII oocytes. DAPI (blue), Spindle (red). Scale bars = 20 μm. **B** Percentages of spindle defects in the Normal, POI, and GEL groups. **C** Representative images of embryos after 120 hours of culture in vitro. Scale bars = 50 μm. **D** The average developmental efficiency of embryos in the Normal, POI, and GEL groups. The percentage was calculated using the number of embryos that reached the blastocyst stage. Developmental rates were statistically analyzed by Student’s t test. **E** Representative image of blastocysts stained with anti-OCT4 (ICM) and DAPI (total cells) in the normal, POI, and GEL groups. Scale bars = 20 μm. **F** (a) ICM and total number of cells in the embryo. (b) The percentage of ICM to total cells in blastocysts. The different superscript letters indicate significant differences (*p* < 0.05); BL, blastocyst; MII, MII stage oocyte; 2C, 2-cell; ICM, inner cell mass

Because more therapeutic effects on ovarian injuries were observed in the GEL group than in the other groups, we further evaluated the effects of this HA gel on reproductive ability. To study the impact of the local delivery of human ESC-MPCs using HA gel on the quality of oocytes in cisplatin-damaged ovaries, all mice were superovulated at 4 weeks after transplantation, and mature oocytes (metaphase II and MII oocytes) were stained with β-tubulin to visualize microtubules in the spindle. Normal mature oocytes had a barrel-shaped spindle apparatus (Fig. 5A, upper panel). The POI group exhibited a significantly higher proportion of abnormal spindle morphology than that in the other groups; however, the GEL group was not significantly different from the normal control group (*p* < 0.05, Fig. 5A, B). Next, we assessed whether the transplantation of human ESC-MPCs with HA gel could improve embryonic development following IVF in a CIP model. The rate of blastocyst formation in all CIP models was still lower than that in the normal control mice, but a significantly higher blastocyst formation rate was observed in the GEL group than in the POI group (Fig. 5C, D). Furthermore, the ICM ratio, which is regarded as an indicator of good-quality embryos and may have high developmental potential [83–86], was significantly higher in the GEL group than in the POI group (Fig. 5E, F). These results indicate that transplantation of human ESC-MPCs with HA gel can affect embryo quality and development.

### Transplantation of human ESC-MPCs with HA gel prevents irregular estrous cycles in CIP mice

**Fig. 6:**
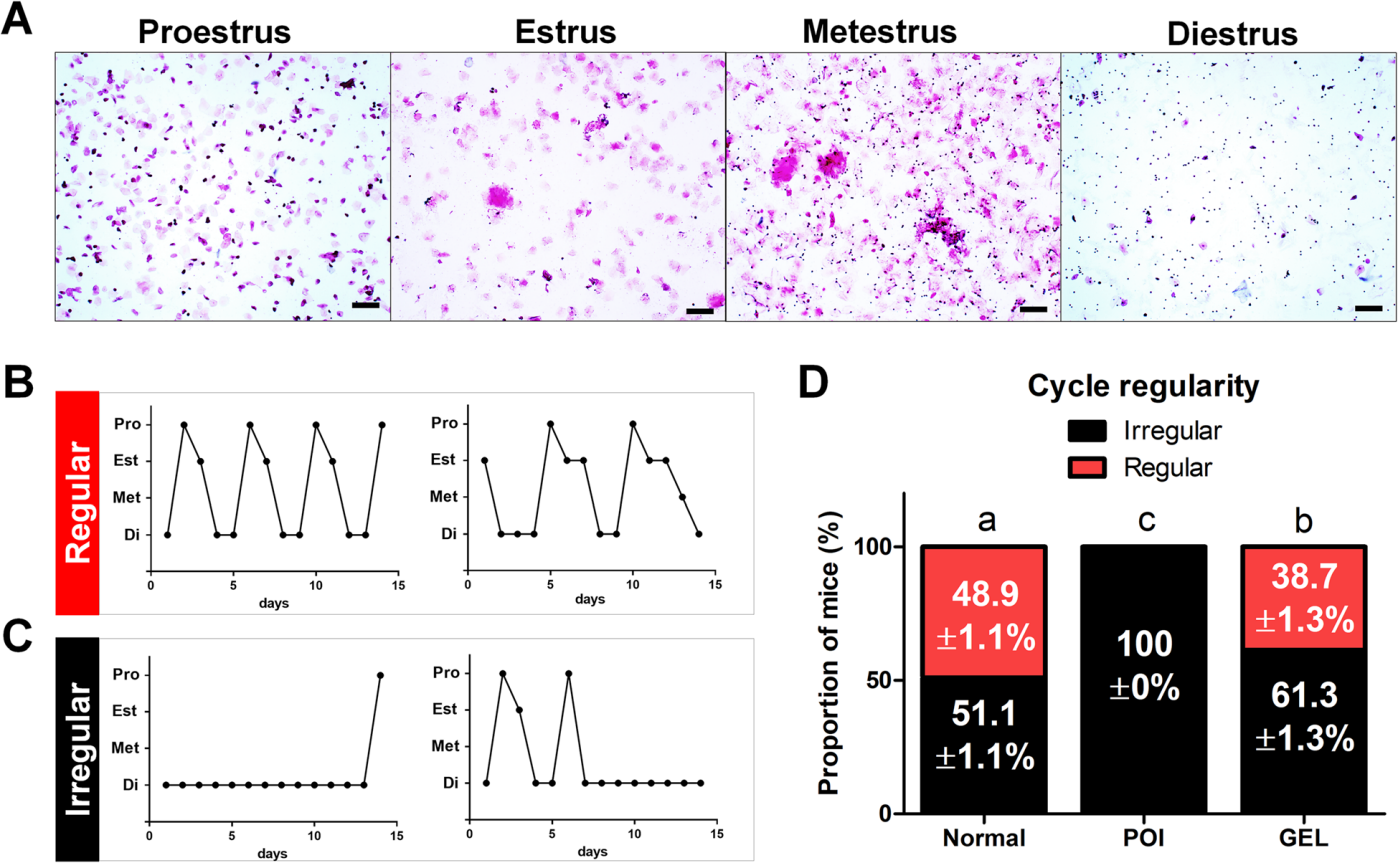
Transplantation of hESC-MPCs with HA gel improved the regularity of the estrous cycle in CIA mice. **A** Representative images of each stage of the estrous cycle. The estrous cycle stage was determined by vaginal cytology. **B**, **C** Representative examples of (**B** regular or **C** irregular) estrous cycling patterns from four individual mice. **D** The percentage of mice with regular (red) or irregular (black) cyclicity was detected at 8 weeks after cell therapy

Chemotherapy-induced POI is accompanied with the prolongation or cessation of the female estrous cycle [5–8]. Thus, to assess the restoration of estrous cycles in the GEL group of CIP mice, estrous cycles were consecutively observed for 2 months after transplantation. The estrous cycle of mice lasts 4–5 days and is divided into four stages (proestrus, estrus, metestrus, and diestrus, Fig. 6A). Regular estrous cycles (Fig. 6B) were repeated every 4–5 days, whereas irregular estrous cycles (Fig. 6C) were prolonged and even remained diestrus for more than 13 days. The average number of regular estrous cycles in the POI group was 0 ± 0%, whereas that in the GEL group was 38.7 ± 1.3% (Fig. 6D). Compared with the GEL group, the POI group had a significantly delayed estrous cycle that stagnated in diestrus for more than 10 days. These results indicate that locally transplanted ESC-MPCs using HA gel could contribute to the prevention of CIA, which is a major indicator of chemotherapy-induced POI.

## Discussion

The present study aimed to evaluate whether locally delivered human ESC-MPCs exert restorative effects in cisplatin-injured mice ovaries. Interestingly, local administration of human ESC-MPCs using HA gel on the backs of mice effectively recovered ovarian structure and function and enhanced the quality of oocytes and embryos in CIP mice. Furthermore, compared with the POI group, the GEL group had significantly recovered body weight and vaginal cyclicity. Thus, our study showed that HA gel can serve as a scaffold for stem cell delivery and may be used in clinical applications to retain fecundity in young cancer survivors.

It is well known that the negative effect of chemotherapy on ovarian functions is a frequent side effect in premenopausal women and can result in POI. Apoptosis of ovarian follicles induced by chemotherapy is strongly related to POI and could induce female infertility, which may be a major consideration for young patients with cancer. Current options for preserving fecundity in patients with cancer include cryopreservation of ovarian tissues, gametes, or embryos prior to anticancer treatment and reduction of gonadal toxicity during chemotherapy [15, 87]. Cryopreservation of oocytes or embryos is the standard option for female patients with cancer. However, these procedures require ovarian stimulation by hormone injections for at least 12 days to collect the follicle and are only used if the patient is postpubertal [88]. Accordingly, these cryopreservation methods are not appropriate in situations requiring urgent anticancer therapy and cannot be used in prepubertal girls. Thus, a better method for preventing chemotherapy-induced POIs is required for these patients.

In our previous study, we evaluated the recovery effects of intravenously delivered human ESC-MPCs in CIP mice [25]. I.V. can enhance pregnancy capacity and can recover ovarian function after chemotherapy, but it still carries a risk of pulmonary embolism and might have low efficiency because of poor viability after injection. The main cause of the low survival rate is anoikis at the injured sites, which is programmed cell death that arises because of a deficit of the ECM [89–92]. The physical structure provided by the ECM is important for cell proliferation and survival because it supports matrix anchorage for cell–cell adhesion [93]. Therefore, we hypothesized that providing such a structure would promote cell survival and improve the efficiency of ESC-MPC functions.

Scaffolds are three-dimensional polymeric biomaterials that provide structural support for tissue regeneration and cell attachment [94]. They can provide structural support for cell adhesion, proliferation, and mechanical properties, which provide mechanical stability and shape to engineered tissues and provide a residence for exogenously applied cells. Moreover, they can be used as delivery vehicles for growth factors, cytokines, and cells to help new tissue formation and remodeling. These can be divided into two types of transplantation methods: implantable scaffolds and injectable scaffolds [95]. Both scaffolds have tunable biocompatibility and degradability, but they have different architectural, biological, and mechanical features. Implantable scaffolds are made of nanofibers, hydrogels, and macroporous scaffolds, which have some benefits in terms of tunable mechanical strength and a variety of fabrication methods, as well as a variety of materials that can be molded to predefined shapes that mimic the natural cell niche. However, these administration routes require invasive surgery and often result in non-homogeneous cell distribution. Injectable scaffolds are colloidal gels made from hydrogels, fibers, microparticles or nanoparticles, and polymers, and those with irregular shapes can freely fill the injured cavity and have a homogeneous cell distribution and can therefore form a uniform tissue. Furthermore, they are minimally invasive and can be directly delivered to the injured sites, which might be suitable for tissue integration.

Classically, scaffolds have been used as artificial ovaries for follicle maturation in vitro or implantation to reduce the risk of contamination of malignant cells and to restore fecundity in the field of oncofertility [56]. Follicles have been isolated from ovaries and sequestered in scaffolds such as fibrin, collagen, alginate, fibrin-alginate, polyethylene glycol, and porcine ovarian extracellular matrix. Transplantation to the peritoneum, ovary, and kidney capsule is performed; therefore, these approaches still have an invasive surgical burden [58, 59, 96–99].

Here, we report for the first time a strategy to restore fertility by simple administration of human ESC-MPCs on the back of CIP mice through intradermal transplantation using implantable or injectable scaffolds. Although Su et al. reported that similar to our strategy, adipose-derived stem cell (ADSC) transplantation by collagen scaffolds restored ovarian function in a rat model of POI, the cells were injected into the core of the ovaries [60]. This method still requires an invasive procedure and carries a risk of ovarian damage resulting from needle puncture. To address these issues, in the present study, we evaluated whether scaffolds would help human ESC-MPCs survive, resulting in improved efficacy of stem cell therapy. After transplantation, residual cells were detected in both retrieved scaffolds from the implanted site, i.e., the back of mice, until 4 weeks after administration (Figure S5). Our previous report showed that I.V.-delivered MPCs can be detected within only 3 days to 2 weeks after injection; therefore, the present study had a 2-fold better survival rate than previous experiments [62]. These results indicate that scaffolds could support structural functions for cell adhesion and increase the survival of human ESC-MPCs over a long period, as monitored by human-specific genomic DNA PCR (hALU, hSRY; Figure S5B) and immunochemistry (STEM121; Figures S5C and D).

Recently, MPC administration has been regarded as a potential therapy for POI that can improve hormone levels, menstrual cycles, pregnancy rates, and the production of live pups [100]. Various cell types, such as bone marrow-derived MPCs, ADSCs, human menstrual blood-derived MPCs, umbilical cord-derived MPCs, endodermal MPCs, and amniotic fluid-derived MPCs, have been used for the treatment of POI. These cells can secrete multiple growth factors and cytokines, such as VEGF, basic FGF2, insulin-like growth factor 1 (IGF1), hepatocyte growth factor, granulocyte colony-stimulating factor (CSF3), keratinocyte growth factor, and transforming growth factor-beta (TGF-β), which play important roles in restoring ovarian function in POI animals [21]. However, the function of secretomes of ESC-MPCs remains unknown. To investigate the therapeutic function of paracrine factors in human ESC-MPCs, we analyzed the components of human ESC-MPC CM. Our results are consistent with those of earlier studies; human ESC-MPCs secreted some of these cytokines, especially high levels of CSF2, CSF3, IGFBP1, IGFBP2, IGFBP3, IGFBP4, IGFBP6, VEGFA, FGF2, PDGFA, PDGFRα, and epidermal growth factor receptor (Figure S4). These factors are well known to regulate cell proliferation and antiapoptosis in ovarian granulosa cells (GCs); in particular, the regulation of local cytokines such as VEGF, FGF2, and IGF can regulate follicular growth if they are absent and fail to grow further [21].

VEGF and FGF2 are angiogenic and antiapoptotic factors that are efficient for inhibiting apoptosis in ovarian grafts, improving the quality of the ovarian cortex, and promoting angiogenesis in the ovary [101, 102]. Interestingly, several reports have shown that using a biomaterial supplemented by VEGF or FGF2 can improve the viability of the ovarian tissue after grafting [102–104]. Consistent with these findings, the present study showed that VEGF and FGF2 secreted by human ESC-MPCs had a positive effect on the restoration of ovarian functions and could reduce apoptosis in ovarian cells in CIP mice (Figures S4 and 4).

CSF2 (also known as granulocyte-macrophage CSF) secreted by the uterine epithelium and placenta plays important roles in reproductive functions, including the development of preimplantation embryos, implantation, and placental development, by regulating the maternal immune response, preventing apoptotic processes, and enhancing glucose uptake [105–108]. It can also be secreted by MPCs and has an antiapoptotic effect; therefore, CSF2 derived from hESC-MPCs might have almost identical roles that could reduce the apoptotic process in injured ovaries (Fig. 4E and F) [109].

The IGFBP family is a carrier of IGFs in the circulation and can be divided into two types of affinities for IGF: low and high. IGFBPs 1, 2, 3, 4, 5, and 6 have a high affinity for IGFBPs and have been proposed to regulate the activities of IGFs by acting as carriers, prolong their half-lives, and directly modulate the interaction of IGF receptors and their interaction [110]. Classically, IGFBPs seem to inhibit IGF by competitive binding to IGF receptors, but in some situations, IGFBPs can potentiate actions for IGF. It was reported that when IGFBP1, IGFBP3, and IGFBP5 binding or adherence increases on the cell surface, IGF1 is released into the cells. IGFBPs could stimulate mitogenesis by helping to release IGF1, which might be another cause of GC proliferation promotion. In rhesus monkeys, androgen treatment appeared to enhance primordial follicle activation through the promotion of IGF1 signaling [111]. Secreted IGFBP from human ESC-MPCs inhibited IGF1 signaling, which might help maintain the ovarian reserve pool (Fig. 3).

AMH is an ovary-specific growth factor and a member of the TGF-β superfamily. Serum levels of this correlate with the size of the ovarian reserve pool and degree of ovarian damage; thus, it might be used as a marker for the diagnosis of females at a risk of POI [79]. Moreover, AMH is a negative regulator of primordial follicle activation that can maintain the ovarian reserve pool. It is not only a diagnostic marker but can also inhibit the burn-out of primordial follicles, which is the current hypothesis for the cause of chemotherapy-induced POI [81, 112]. AMH is initially secreted in GCs of primary follicles, and the secreted level increases until the antral follicular stage. Chemotherapy can induce the death of these growing follicles, leading to a reduction in AMH levels. In this study, we detected the gene and protein expression of AMH in the ovary after cisplatin injection to measure the degree of recovery of growing follicles. The HA gel and Sponge groups had significantly higher levels of AMH than the other three groups (I.V., I.D., and POI; Fig. 4A, B), suggesting that both methods enhanced the efficacy of human ESC-MPCs in reducing cisplatin-induced death of growing follicles. Furthermore, these results indicate that the secretome of human ESC-MPCs could be successfully delivered to the ovaries through circulating systems that may influence the ovarian niche and protect against the ovotoxicity of cisplatin. In particular, the HA gel group had significantly reduced apoptotic activity and increased estrogen levels compared with those in any other CIP group treated with cell therapy (Fig. 4C, E, F). This might be because HA gel is better suited for the rapid diffusion of paracrine factors than sponge-type scaffolds, which could make human ESC-MPCs more likely to protect against chemotherapy-induced ovarian damage [54]. These results showed that the secretomes of human ESC-MPCs and structural support of scaffolds for long-term survival may play the most effective roles in stem cell therapy, rather than directly affecting cells that can enter injured tissues.

As the HA gel group had the best recovered ovarian functions, we demonstrated that the fertility functions in the HA gel group were improved compared with those in the POI group by evaluating the quality of oocytes and embryos (Fig. 5). In addition, the HA gel group showed a more regular estrous cycle than that in the POI group at 2 months after transplantation, which may indicate that the HA gel method could be used as a long-term treatment against POI and fertility preservation (Fig. 6). Amenorrhea and oligomenorrhea, which are caused by estrogen deficiency, are major symptoms of POI and are suggested to provide direct evidence for the diagnosis of POI [15]. Recent literature has reported that menstrual function patterns are related to diverse diseases, such as decreased lung function, breast and ovarian cancer, cardiovascular disease, bone fracture, and osteoporosis, as well as to the risk of death [113–123]. Therefore, patients with POI who have CIA may need long-term medical follow-up and constant care to maintain their lifelong health. Hormone replacement therapy is ordinarily used in patients with POI to prevent or reduce these symptoms, but it can increase the risk of developing breast cancer, endometrial cancer, endometrial hyperplasia, stroke, and venous thromboembolism [124]. Thus, it is important to develop a proper treatment method for CIA after chemotherapy to avoid these side effects and possible long-term sequelae. In the present study, the HA gel group successfully maintained their estrous (reproductive) cycles for a long time, indicating that locally delivered human ESC-MPCs with a scaffold of HA gel may be a novel option for the treatment of CIA. Moreover, this method could help increase the quality of life and fertility preservation after cancer survival in young patients.

## Conclusions

To the best of our knowledge, this is the first report on the local transplantation of human ESC-MPCs using scaffolds into easily accessible regions, which could effectively restore ovarian functions and female fecundity in cisplatin-induced POI. The use of a scaffold can increase the retention of human ESC-MPCs in vivo for a long time; therefore, it could provide more effective therapeutic functions to cells for the treatment of various diseases. Our study provides new insights into the administration methods of stem cell therapy. The human ESC-MPC/scaffold method could be a clinically promising and safe method to reduce chemotherapy-induced POI.

### Supplementary information

Supplementary information which included four figures.

## Supplementary Information

**Additional file 1: Figure S1.** Physical properties of the scaffolds. (A) The spectra of attenuated total reflection−Fourier transform infrared and (B) thermogravimetric analysis. **Figure S2**. Rheological analysis of HA 100% and 50% gel. The dynamic (A) storage modulus (G‘), (B) loss modulus (G“) and (C) loss tangent (tanδ) values plotted against the frequency of the HA gels at 37°C. **Figure S3**. Negative control and positive control for Figure 1E. The negative control of two types of scaffolds was only stained in the bare gel and sponge without cells to verify its background. The positive control was stained for the ESC-MPCs seeded scaffolds after intentionally inducing apoptosis using hydrogen peroxide (All experiments were conducted with the same staining time, fluorescence exposure, and gain value for each staining dye). **Figure S4.** A growth factor array was performed to detect paracrine factors in the conditioned medium of human ESC-MPCs. (A) The map of the growth factor array provided by the manufacturer. (B) Representative fluorescence images of the growth factor assay in human ESC-MPC conditioned medium cultured for 6 and 24 hours. Secreted factors were arrayed on a glass chip containing 41 different growth factor antibodies and detected with microarray scanner. (C) Selective map of human ESC-MPC-enriched growth factors. (D) The signals were quantified by densitometry and the expression in basal medium was set as the control. (E) Highly enriched proteins in human ESC-MPC-conditioned medium were categorized by biological process using Gene Ontology (GO) enrichment analysis in EnrichR. (F) Enriched KEGG pathways in the human ESC-MPC-conditioned medium. **Figure S5.** Tracking of human ESC-MPCs *in vivo* in transplanted scaffolds. (A) Residual scaffolds (a, Sponge; b, GEL) were stained with H&E at 1 week after implantation. Scale bars=1000μm (B) Expression of the human-specific ALU sequence and SRY gene in residual scaffolds engrafted with human ESC-MPCs, as assessed by gDNA-PCR. (C, D) The residual human ESC-MPCs were stained with the human cytoplasmic marker, stem121, and DAPI in scaffolds at 1 and 4 weeks after implantation. Scale bars=20μm

## Data Availability

The authors declare that the dataset supporting the conclusions of this study is included within the article and its supplementary information files.

## Abbreviations

POI: Premature ovarian insufficiency
ESC: Embryonic stem cell
MPC: Mesenchymal progenitor cell
PLGA: Poly(D, L-lactide-co-glycolide)
MH: Magnesium hydroxide (Mg(OH)_2_)
HA: Hyaluronic acid
CIA: Chemotherapy-induced amenorrhea
I.V.: Intravenous
GEL: Hydrogel
FDA: Food and Drug Administration
TGA: Thermogravimetric analyzer
ATR-FTIR: Attenuated total reflection-Fourier transform infrared
LVE: Linear viscoelastic
DMEM: Dulbecco’s modified Eagle’s medium
CIP: Cisplatin-induced POI
WT: Wild type
AMH: Anti-Müllerian hormone
ELISA: Enzyme-linked immunosorbent assay
PMSG: Pregnant mare serum gonadotropin
hCG: Human chorionic gonadotropin
IHC: Immunohistochemistry
CM: Conditioned medium
I.D.: Intradermal
ZPR: Zona pellucida remnant
ICM: Inner cell mass
ECM: Extracellular matrix
ADSC: Adipose-derived stem cell
VEGF: Vascular endothelial growth factor
FGF2: Fibroblast growth factor
IGF1: Insulin-like growth factor 1
HGF: Hepatocyte growth factor
CSF: Colony-stimulating factor
KGF: Keratinocyte growth factor
TGF-β: Transforming growth factor-beta
IGFBPs: Insulin-like growth factor binding proteins
PDGF: Platelet-derived growth factor
EGFR: Epidermal growth factor receptor
GC: Granulosa cell
HRT: Hormone replacement therapy
