# Supplementary material for: Prevention of chemotherapy-induced premature ovarian insufficiency in mice by scaffold-based local delivery of human embryonic stem cell-derived mesenchymal progenitor cells
Source: Stem Cell Res Ther. 2021 Jul 31;12:431. doi: 10.1186/s13287-021-02479-3 (PMC8325282; doi:10.1186/s13287-021-02479-3)
Supplement: Supplementary file 1 — Additional file 1: Figure S1. Physical properties of the scaffolds. (A) The spectra of attenuated total reflection−Fourier transform infrared and (B) thermogravimetric analysis. Figure S2. Rheological analysis of HA 100% and 50% gel. The dynamic (A) storage modulus (G‘), (B) loss modulus (G“) and (C) loss tangent (tanδ) values plotted against the frequency of the HA gels at 37°C. Figure S3. Negative control and positive control for Figure 1E. The negative control of two types of scaffolds was only stained in the bare gel and sponge without cells to verify its background. The positive control was stained for the ESC-MPCs seeded scaffolds after intentionally inducing apoptosis using hydrogen peroxide (All experiments were conducted with the same staining time, fluorescence exposure, and gain value for each staining dye). Figure S4. A growth factor array was performed to detect paracrine factors in the conditioned medium of human ESC-MPCs. (A) The map of the growth factor array provided by the manufacturer. (B) Representative fluorescence images of the growth factor assay in human ESC-MPC conditioned medium cultured for 6 and 24 hours. Secreted factors were arrayed on a glass chip containing 41 different growth factor antibodies and detected with microarray scanner. (C) Selective map of human ESC-MPC-enriched growth factors. (D) The signals were quantified by densitometry and the expression in basal medium was set as the control. (E) Highly enriched proteins in human ESC-MPC-conditioned medium were categorized by biological process using Gene Ontology (GO) enrichment analysis in EnrichR. (F) Enriched KEGG pathways in the human ESC-MPC-conditioned medium. Figure S5. Tracking of human ESC-MPCs in vivo in transplanted scaffolds. (A) Residual scaffolds (a, Sponge; b, GEL) were stained with H&E at 1 week after implantation. Scale bars=1000μm (B) Expression of the human-specific ALU sequence and SRY gene in residual scaffolds engrafted with human ESC-MPCs, as assessed by gDN [file 13287_2021_2479_MOESM1_ESM.docx]

**Prevention of chemotherapy-induced premature ovarian insufficiency in mice by scaffold-based local delivery of human embryonic stem cell-derived mesenchymal progenitor cells**

Eun-Young Shin ^1,4^, Da-Seul Kim ^2,4^, Min Ji Lee ^1^, Ah Reum Lee ^3^, Sung Han Shim ^1^, Seung Woon Baek ^1^, Dong Keun Han ^1, #^, and Dong Ryul Lee ^1, #^

^1^ Department of Biomedical Science, CHA University, 335 Pangyo-ro, Bundang-gu, Seongnam-si, Gyeonggi 13488, Republic of Korea

^2^ School of Integrative Engineering, Chung-Ang University, 84 Heukseok-ro, Dongjak-gu, Seoul, 06974, Republic of Korea

^3^ CHA Advanced Research Institute, CHA hospital, 335 Pangyo-ro, Bundang-gu, Seongnam-si, Gyeonggi 13488, Republic of Korea

^4^ Co-first author

^#^ Co-correspondence: [drleedr@cha.ac.kr](mailto:drleedr@cha.ac.kr) (D.R.L) and [dkhan@cha.ac.kr](mailto:dkhan@cha.ac.kr) (D.K.H)

Running Head: prevention of chemotherapy-induced POI by ESC-MPCs


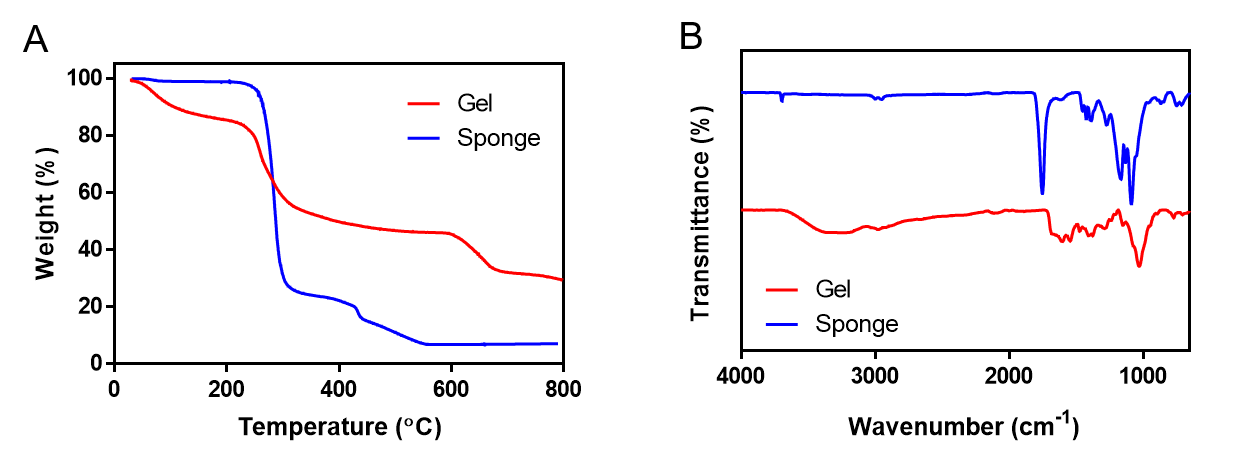


**Figure S1.** Physical properties of the scaffolds. (A) The spectra of attenuated total reflection−Fourier transform infrared and (B) thermogravimetric analysis.


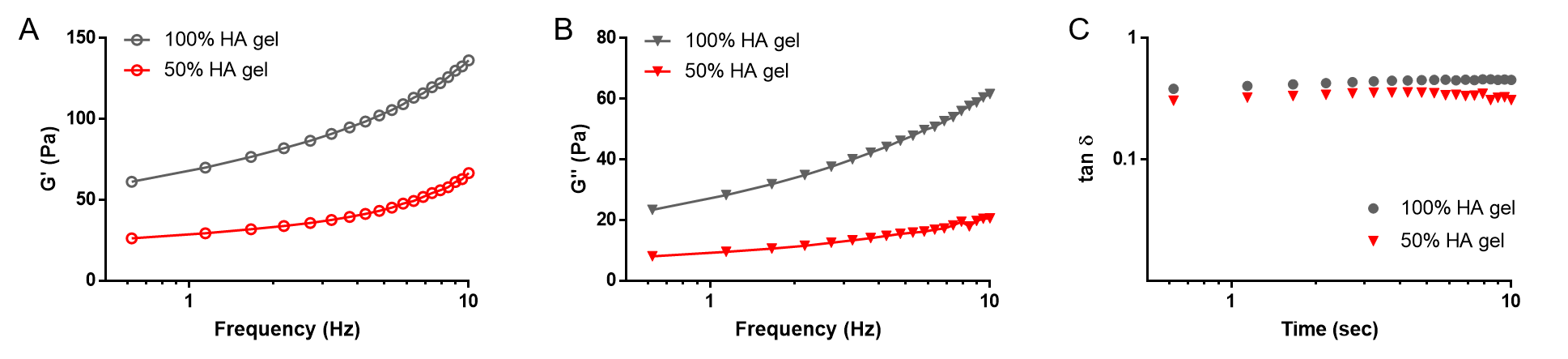


**Figure S2**. Rheological analysis of HA 100% and 50% gel. The dynamic (A) storage modulus (G‘), (B) loss modulus (G“) and (C) loss tangent (tanδ) values plotted against the frequency of the HA gels at 37℃.


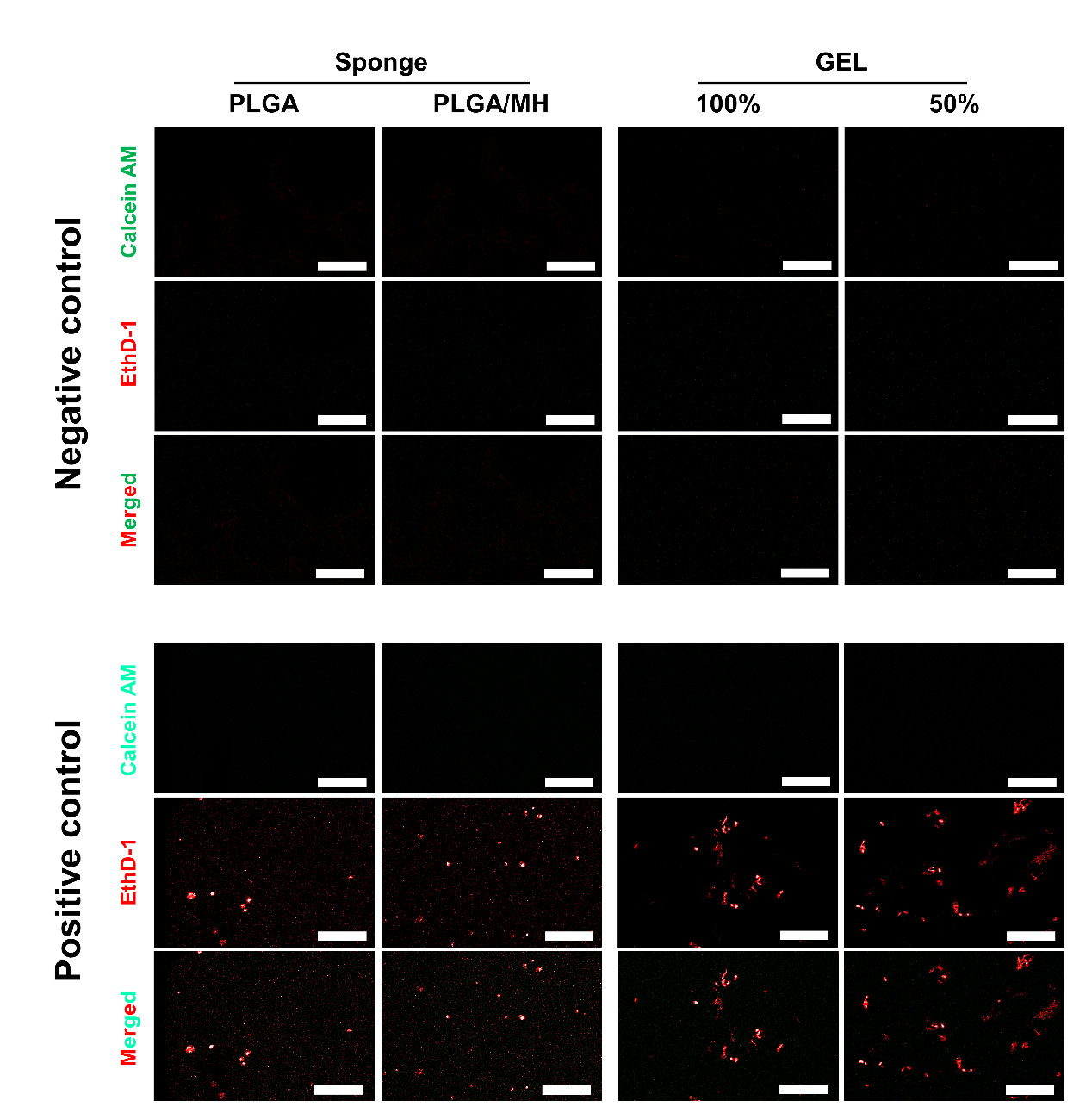


**Figure S3**. Negative control and positive control for Figure 1E. The negative control of two types of scaffolds was only stained in the bare gel and sponge without cells to verify its background. The positive control was stained for the ESC-MPCs seeded scaffolds after intentionally inducing apoptosis using hydrogen peroxide (All experiments were conducted with the same staining time, fluorescence exposure, and gain value for each staining dye).


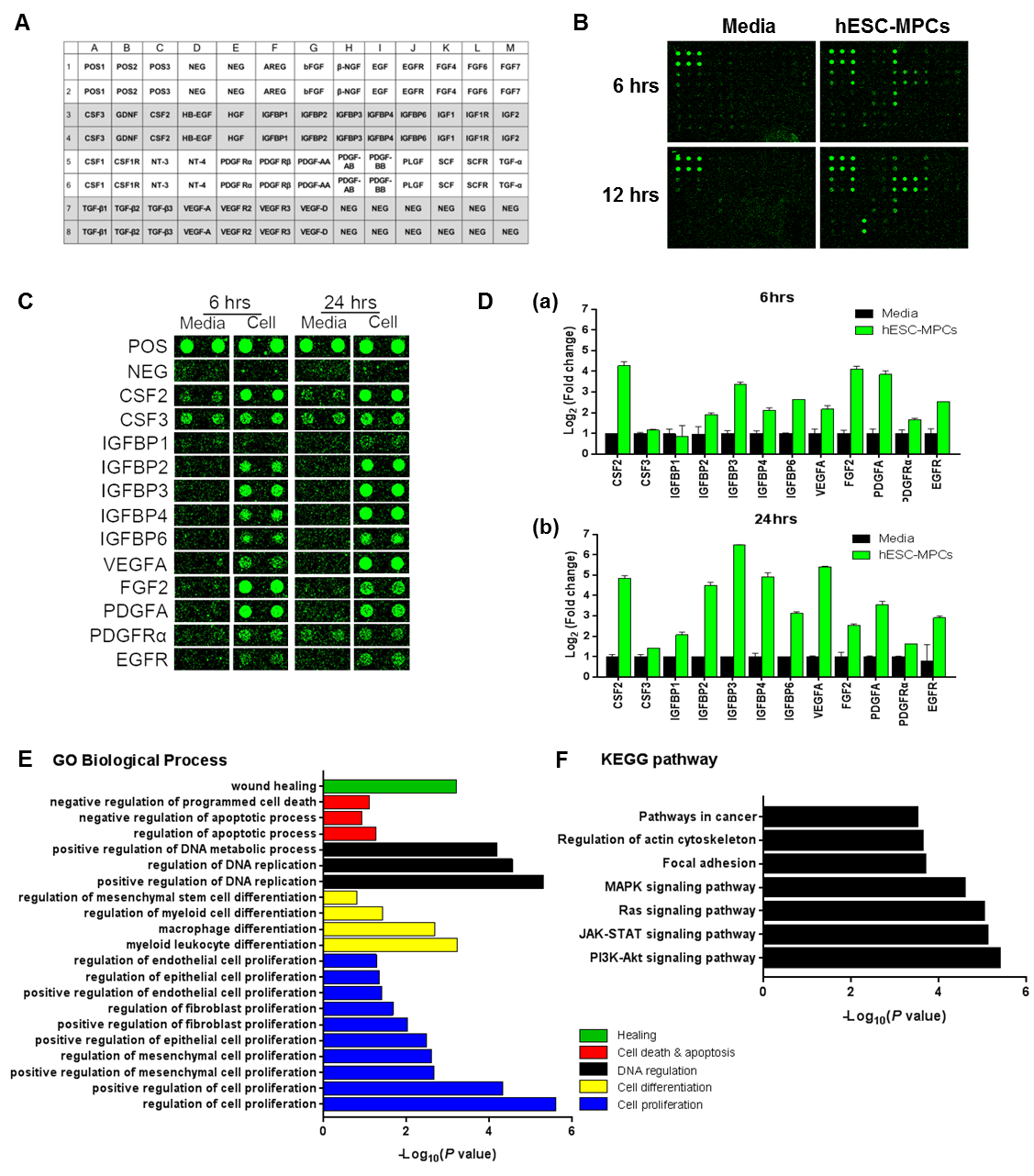


**Figure S4.** A growth factor array was performed to detect paracrine factors in the conditioned medium of human ESC-MPCs. (A) The map of the growth factor array provided by the manufacturer. (B) Representative fluorescence images of the growth factor assay in human ESC-MPC conditioned medium cultured for 6 and 24 hours. Secreted factors were arrayed on a glass chip containing 41 different growth factor antibodies and detected with microarray scanner. (C) Selective map of human ESC-MPC-enriched growth factors. (D) The signals were quantified by densitometry and the expression in basal medium was set as the control. (E) Highly enriched proteins in human ESC-MPC-conditioned medium were categorized by biological process using Gene Ontology (GO) enrichment analysis in EnrichR. (F) Enriched KEGG pathways in the human ESC-MPC-conditioned medium.


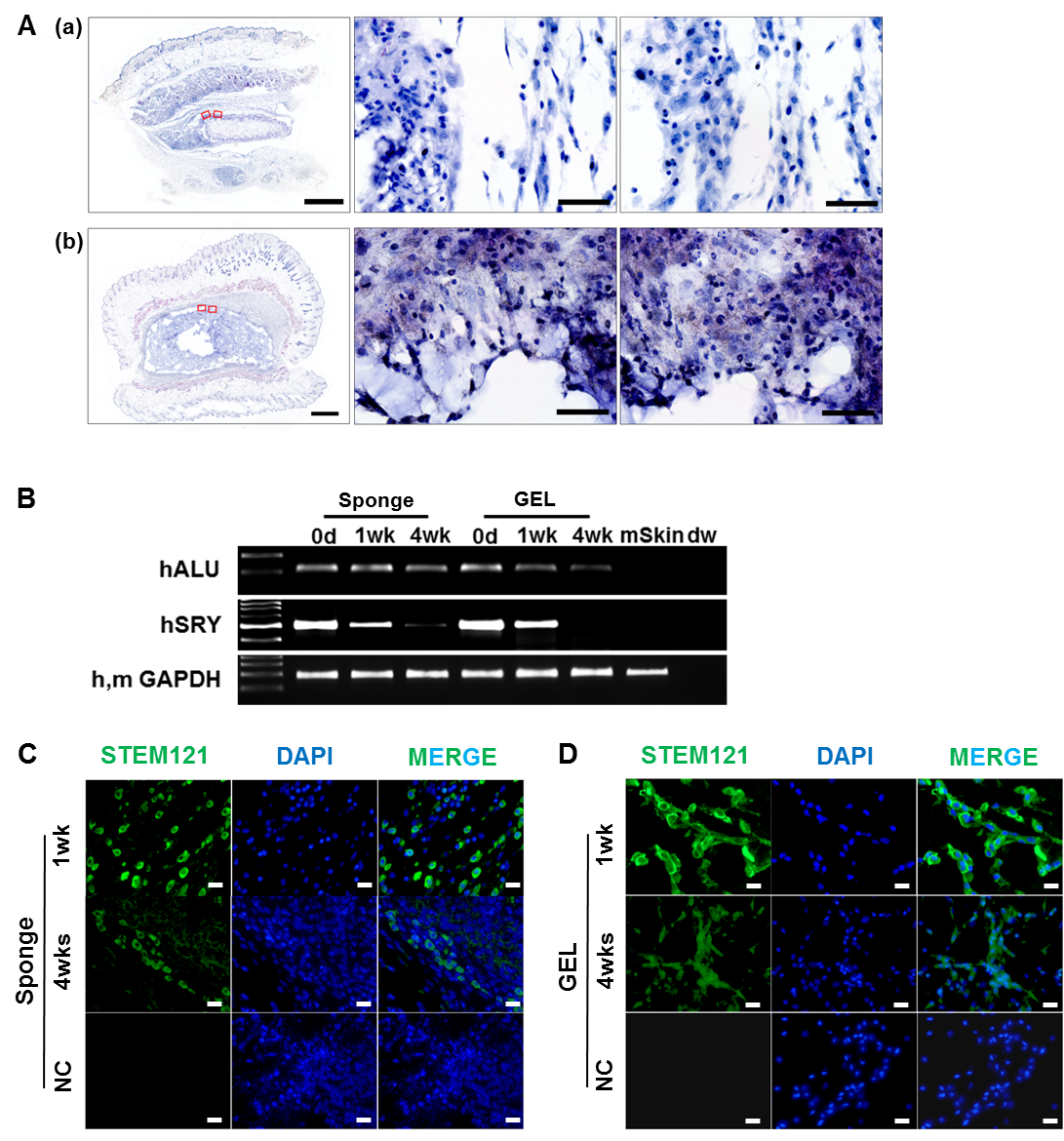


**Figure S5.** Tracking of human ESC-MPCs *in vivo* in transplanted scaffolds. (A) Residual scaffolds (a, Sponge; b, GEL) were stained with H&E at 1 week after implantation. Scale bars=1000μm (B) Expression of the human-specific ALU sequence and SRY gene in residual scaffolds engrafted with human ESC-MPCs, as assessed by gDNA-PCR. (C, D) The residual human ESC-MPCs were stained with the human cytoplasmic marker, stem121, and DAPI in scaffolds at 1 and 4 weeks after implantation. Scale bars=20μm
